# Genetic diversity and haplotype analysis of cattle hydatid cyst isolates using mitochondrial markers in Kazakhstan

**DOI:** 10.14202/vetworld.2024.763-770

**Published:** 2024-04-07

**Authors:** Rabiga Uakhit, Sofiya Yalysheva, Aida Abdybekova, Ainura Smagulova, Lyudmila Lider, Karina Jazina, Aidana Tautanova, Vladimir Kiyan

**Affiliations:** 1 Laboratory of Biodiversity and Genetic Resources, National Center for Biotechnology, Astana, Kazakhstan; 2 Laboratory of Parasitology, Department of Veterinary Medicine, S. Seifullin Kazakh Agrotechnical Research University, Astana, Kazakhstan; 3 Department of Molecular Biology and Genetics, al-Farabi Kazakh National University, Almaty, Kazakhstan; 4 Laboratory of Parasitology and Mycology, Kazakh Scientific Research Veterinary Institute, Almaty, Kazakhstan; 5 Department of Microbiology and Virology, Astana Medical University, Astana, Kazakhstan; 6 Department of Scientific and Analytical Work, West Kazakhstan Marat Ospanov Medical University, Aktobe, Kazakhstan

**Keywords:** cattle, cystic echinococcosis, *Echinococcus granulosus*, genotypes, haplotype, Kazakhstan

## Abstract

**Background and Aim:**

In Kazakhstan, the study of *Echinococcus* infection among farm animals is crucial to monitor the invasion among livestock and map the data obtained. Unfortunately, there are only partial data on the study of *Echinococcus* among cattle’s in Kazakhstan, which makes it difficult to conduct a comparative analysis of the epidemiological situation among livestock animals. The present study aimed to molecularly identify the species and haplotypes of the *E. granulosus* complex infecting cattle in Kazakhstan and investigate their genetic variation relative to mitochondrial (mt) targets.

**Materials and Methods:**

Individual cyst isolates (n = 700) were collected from infected cattle lungs and livers after slaughter from the slaughterhouse. Total DNA was extracted from the germinal layers of the cyst from each isolate. This DNA sequenced partial mt genes of cytochrome c oxidase 1 (450 bp) and NADH dehydrogenase 1 (1200 bp).

**Results:**

We determined that all the sequences were detected as *E. granulosus* s.s., of which 69 (94.5%) samples belonged to G1, and only 4 (5.4%) samples belonged to the G3 genotype. After bioinformatic analysis, 38 haplotypes were identified.

**Conclusion:**

Our findings revealed that the G1 genotype of *E. granulosus* s.s. is the predominant cattle genotype in Kazakhstan. However, only one region showed the presence of two genotypes G1 and G3, in the sequence, which suggests that further research is needed to investigate the epidemiology of *Echinococcus* infection in cattle in Kazakhstan.

## Introduction

Cystic echinococcosis (CE) is a chronic helminthic zoonosis with a worldwide distribution. CE affects a wide variety of livestock species acting as intermediate hosts (IHs), whereas humans represent aberrant hosts of IHs [1]. *Echinococcus* parasites require carnivores as final hosts and herbivores as IHs to complete their life cycle. The adult stage inhabits the small intestine of a carnivore and produces hundreds of worms that shed thousands of infected eggs in the stool of the host. As soon as the IH ingests the larvae, they move through the bloodstream to the internal organs, where they form fluid-filled cysts. These cysts may develop into thousands of protoscolices that can mature into adult worms if they are ingested by a definitive host. Human beings are accidental hosts that do not perpetuate the life cycle.

Thus, to date, the *Echinococcus granulosus* complex comprises five species: *E. granulosus sensu stricto* (s.s.) (clustering genotypes G1, G2, and G3), *Echinococcus equinus* (G4), *Echinococcus ortleppi* (G5), *Echinococcus canadensis* (grouping genotypes G6–G8 and G10), and *Echinococcus felidis* (G9) [2–5]. *E. granulosus* s.l. exhibits a high genotypic diversity with 10 genotypes (G1–G10) that have been molecularly distinguished so far, predominantly based on genetic polymorphism of mitochondrial (mt) genes [6, 7]. *E. granulosus* s.s. (genotypes G1, G3) is the most wide-spread species of the *E. granulosus* s.l. complex. In general, reported mt haplotypes are gathered by similarity around G1 or G3, whereas G2 seems to be a micro-variant of G1 or often of G3 [8, 9]. To date, data on the molecular typing of genotyping and haplotypes of *E. granulosus* complex circulating in Kazakhstan have not been published.

This study aimed to molecularly identify species and haplotypes of the *E. granulosus* complex infecting cattle in Kazakhstan and to investigate their genetic variation respective to mt targets. Kazakhstan is considered highly endemic to hydatid cysts in humans and animals.

## Materials and Methods

### Ethical approval

Samples of *Echinococcu*s cysts from cattle were collected from the slaughterhouse and delivered to the parasitological laboratory of the Faculty of Veterinary Medicine. This study was approved by the Animal Ethics Committee of the National Center for Biotechnology (protocol No. 1 dated April 01, 2022). All animal experiments were conducted in accordance with the World Medical Association Code of Ethics (Declaration of Helsinki) (http://ec.europa.eu/environment/chemicals/lab_animals/legal_en.htm).

### Study period and location

The study was conducted from August 2022 to October 2023, in the laboratory of parasitology of the Seifullin Kazakh Agrotechnical University and the laboratory of biodiversity and genetic resources of the National Center of Biotechnology, located in Astana, Kazakhstan. Cattle samples were collected from 17 regions of Kazakhstan (Kostanay, Akmola, South Kazakhstan, Pavlodar, Karaganda, East Kazakhstan, West Kazakhstan, Aktobe, Atyrau, Ulytau, North-Kazakhstan, Almaty, Kyzylorda, and Mangystau regions).

### DNA extraction and polymerase chain reaction (PCR) analysis

The cysts showed different conditions; some were calcified and others purulent. As a result, the most suitable samples were selected for future studies. DNA was successfully extracted from 73 cysts using a GeneJet genomic DNA purification kit (Thermo Fisher, USA, Cat.: K0701) with some modifications. Briefly, *Echinococcus* cysts extracted from organs were homogenized in Eppendorf tubes using a pestle, lyses buffer, and Proteinase K.

PCR was applied to identify the genetic diversity of *Echinococcus* spp. using two primer pairs targeting cytochrome c oxidase subunit 1 (cox1: forward 5′-TTTTTTGGGCATCCTGAGGTTTAT-3′ and reverse 5′-TAAAGAAAGAACATAATGAAAATG-3′) and dehydrogenase subunit 1 (nad1: forward 5′-TGGAACTCAGTTTGAGCTTTACTA-3′ and reverse 5′-ATATCAAAGTAACCTGCTA TGCAG-3′) [4, 10]. Reactions were performed in 15 μL 2^×^ GoTaq Hot Start MasterMix, 9 μL nuclease-free water, 1 μL total primers, and 2.5 μL extracted DNA.

### Electrophoresis and sequencing

Agarose gels (1%) were prepared in 1× TBE solution containing 8 ng/µL ethidium bromide (Sigma, E1510). Electrophoresis was performed for 50 min at 120 V using 10 μL PCR products with a DirectLoad 100 bp Low ladder ready-to-use (Sigma, D3687-1VL). The PCR-amplified target gene fragment was purified according to the manufacturer’s protocol using a QIAquick PCR Purification Kit, (Qiagen, Germany, Cat.: 28106). Sequencing was performed using the Seq Studio Genetic Analyzer (Thermo Fisher Scientific Applied Biosystems, USA) according to the manufacturer’s instructions. The resulting nucleotide sequences were visually checked using BioEdit version 7.0. BLAST was used to compare the nucleotide sequences of the studied species with those of other sequences in the National Center for Biotechnology Information (NCBI) GenBank database. Nucleotide sequences of the studied species were deposited in the NCBI GenBank database.

### Phylogenetic analysis

Nucleotide sequences obtained in the current study were submitted to GenBank and published under accession numbers for cox1 gene and nad1 (Table-1). The obtained sequences were manually edited, and sequence similarity searches compared to the GenBank reference sequences were performed using BLAST (https://blast.ncbi.nlm.nih.gov). Nucleotide sequences for cox1 and nad1 partial genes were aligned with the MUSCLE multiple sequence alignment program. Phylograms were constructed using a concatenated dataset with the MEGA11 software [11] using the maximum likelihood (ML) method. *Mesocestoides* spp. (MH998121) was used as an out-group.

**Table-1:** Cattle samples of *Echinococcus* spp. submitted to GenBank.

Geographical origin	Isolate	GB number (co×1)	GB number (nad1)	Genotype	Haplotype
Akmola region	3-22-13	OR135798	OR294861	G1	hp1
	3-22-14	OR136140	OR294862	G1	hp2
	3-22-15	OR136142	OR294863	G1	hp3
	3-22-16	OR136143	OR294864	G1	hp1
	3-22-17	OR136144	OR294865	G1	hp4
Aktobe region	4-22-2	OR136163	OR294866	G3	hp5
	4-22-3	OR136184	OR294867	G1	hp6
	4-22-4	OR136190	OR294868	G1	hp5
	4-22-5	OR140728	OR294869	G3	hp7
	4-22-6	OR140736	OR294870	G3	hp7
	4-22-7	OR140912	OR294871	G3	hp8
	4-22-8	OR140735	OR294872	G1	hp9
	4-22-9	OR140738	OR294873	G1	hp5
Almaty	5-22-4	OR136223	OR294874	G1	hp10
	5-22-5	OR136294	OR294875	G1	hp10
	5-22-6	OR136364	OR294876	G1	hp11
	5-22-7	OR136456	OR294877	G1	hp12
	5-22-8	OR136369	OR294878	G1	hp13
Atyrau	6-22-1	OR136380	OR294879	G1	hp5
	6-22-2	OR136382	OR294880	G1	hp5
	6-22-3	OR136455	OR294881	G1	hp5
	6-22-4	OR136384	OR294882	G1	hp5
	6-22-5	OR136386	OR294883	G1	hp14
West-Kazakhstan	7-22-1	OR136387	OR294884	G1	hp5
	7-22-2	OR136397	OR294885	G1	hp15
	7-22-3	OR136457	OR294886	G1	hp16
	7-22-4	OR139966	OR294887	G1	hp15
	7-22-5	OR139967	OR294888	G1	hp15
Dzhambul	8-22-9	OR136471	OR294889	G1	hp5
	8-22-10	OR136492	OR294890	G1	hp15
	8-22-11	OR136494	OR294891	G1	hp5
	8-22-12	OR136509	OR294892	G1	hp17
	8-22-13	OR136513	OR294893	G1	hp17
Karagandy	9-22-11	OR140739	OR294894	G1	hp18
	9-22-12	OR140741	OR294895	G1	hp5
	9-22-13	OR140744	OR294896	G1	hp19
	9-22-14	OR140778	OR294897	G1	hp5
	9-22-15	OR140783	OR294898	G1	hp20
Kostanay	10-22-1	OR140785	OR294899	G1	hp5
	10-22-2	OR140786	OR294900	G1	hp21
	10-22-3	OR140788	OR294901	G1	hp22
	10-22-4	OR140819	OR294902	G1	hp23
	10-22-5	OR140821	OR294903	G1	hp23
Kyzylorda	11-22-5	OR140830	OR294904	G1	hp24
	11-22-6	OR140827	OR294905	G1	hp25
	11-22-7	OR140824	OR294906	G1	hp26
	11-22-8	OR140833	OR294907	G1	hp27
	11-22-9	OR342713	OR294908	G1	hp29
Mangystau	12-22-16	OR140847	OR294909	G1	hp15
	12-22-19	OR140849	OR294910	G1	hp5
	12-22-24	OR140837	OR294911	G1	hp28
	12-22-25	OR140852	OR294912	G1	hp5
	12-22-26	OR140840	OR294913	G1	hp5
South-Kazakhstan region	13-22-1	OR140853	OR294914	G1	hp5
	13-22-2	OR140855	OR294915	G1	hp5
	13-22-3	OR140856	OR294916	G1	hp5
	13-22-4	OR140857	OR294917	G1	hp29
	13-22-5	OR140890	OR294918	G1	hp30
Pavlodar	14-22-5	OR140869	OR294919	G1	hp4
	14-22-9	OR140862	OR294920	G1	hp5
	14-22-11	OR140866	OR294921	G1	hp5
	14-22-12	OR140867	OR294922	G1	hp31
	14-22-13	OR140870	OR294923	G1	hp32
North-Kazakhstan region	15-22-14	OR140877	OR294924	G1	hp33
	15-22-15	OR140880	OR294925	G1	hp33
	15-22-16	OR140893	OR294926	G1	hp5
	15-22-17	OR140910	OR294927	G1	hp34
	15-22-18	OR140891	OR294928	G1	hp35
East-Kazakhstan region	16-22-16	OR140892	OR294929	G1	hp36
	16-22-17	OR140899	OR294930	G1	hp12
	16-22-18	OR140898	OR294931	G1	hp37
	16-22-19	OR140906	OR294932	G1	hp38
	16-22-21	OR140911	OR294933	G1	hp5

co×1 = Cytochrome c oxidase subunit 1

### Haplotype analysis

The haplotype data file was generated using the DnaSP v.6 software. Statistical parsimony networks were used to analyze haplotype genealogy in cox1 and nad1 concatenated datasets using TCS (Statistical Parsimony by A. R. Templeton, K. A. Crandall, and C. F. Sing) implemented in PopART software [12]. We constructed the networks with a 95% probability limit. To distinguish between synonymous and non-synonymous mutations, nucleotide sequence translation was performed using the DnaSP v.6 software (http://www.ub.edu/dnasp/downloadTv6.html).

## Results

### Characterization of mtDNA haplotypes

Throughout the study, 700 hydatid cyst samples were meticulously collected from 14 distinct regions, with an average of 3.7 cysts per host. Subsequently, DNA extraction was successfully performed on 76 samples, and sequencing was performed on each of the selected samples. All cysts were identified as *E. granulosus* s.s. with 38 identified haplotypes among our datasets, including two concatenated mt gene markers: cox I and nad I.

The final aligned sequences with positions 1100–1400 were converted in FASTA and NEXUS format for further analysis using MEGAX, DnaSP v.6, PopArt v. 1.7 software. Point mutations in the sequences were identified after the alignment. Table-1 shows the nucleotide variation positions based on reference sequences. To evaluate the relationships among the different *E. granulosus* s.s. haplotypes, a network of mt haplotypes based on the concatenated sequences was constructed (Table-1).

### Phylogenetic analysis

These samples were subjected to bioinformatic analysis, and the resulting sequences were deposited on the NCBI GenBank platform for further reference.

The obtained sequences were manually edited and sequence similarity searches compared to the GenBank reference sequences were performed using BLAST (https://blast.ncbi.nlm.nih.gov/BLAST/). Nucleotide sequences for concatenated cox1 and nad1 partial genes were aligned with the MUSCLE multiple sequence alignment algorithms.

**Figure-1:**
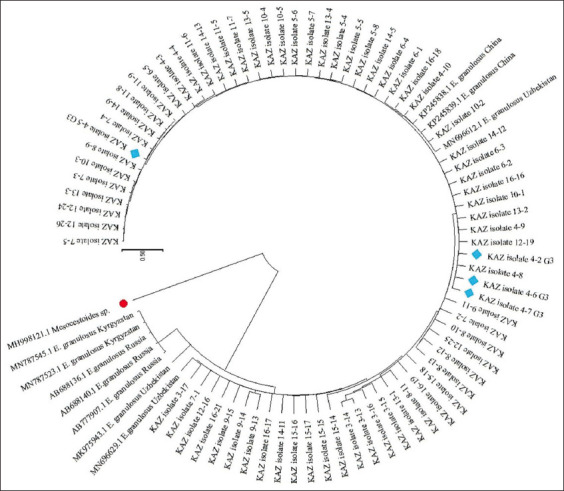
Maximum Likelihood phylogenic tree from concatenated sequences of partial genes cox1 and nad1 showing relationships between the examined Kazakhstan isolates and GenBank retrieved related sequences of *E. granulosus* from neighborhood countries. (*outgroup marked with red dot, *E. granulosus* studied in this research belong to the genotype G3 marked with blue sing). *E. granulosus=Echinococcus granulosus*.

Analysis of the concatenated sequences of the two partial genes revealed that all samples belonging to *E. granulosus* were grouped together. A tree was rooted with an out-group by *Mesocestoides* spp. to produce this grouping (Figure-1).

A set of 84 nucleotide sequences was analyzed using the ML method and the Tamura-Nei model [11] to analyze the evolutionary history of a set of the resulting tree diagram, drawn on a scale, displays the relationships between sequences with branch lengths measured in terms of the number of substitutions per site. A tree with the highest log likelihood (11369.25) is presented, and the proportion of sites where at least one unambiguous base is present in at least one sequence for each descendent clade is indicated next to every internal node in the tree. The final dataset contained a total of 2551 positions.

Various point mutations were identified and analyzed after aligning the sequences. Table-2 summarizes the study findings, which highlight the specific positions of nucleotide variations observed in analyzed sequences.

**Table-2:** Nucleotide variation positions of concatenated of the mt co×1 and nad1 genes among 38 analyzed haplotypes.

Nucleotide position	709	710	735	736*	737	738	741	742	743	750	757	852	853	854	856*	875	876	887	944	975	986	999	1002*	1047	1049
Hp 1	A	A	C	C	A	A	T	C	G	A	T	C	G	A	G	G	T	A	C	T	C	T	T	C	C
Hp 2	.	.	.	.	.	.	.	.	.	.	.	A	.	.	.	.	.	.	.	C	.	.		.	.
Hp 3	.	.	.	.	.	.	.	.	.	.	.	.	.	.	.	.	.	.	T	.	T	.		A	A
Hp 4	C	.	.	.	.	.	.	.	.	.	.	A	.	.	.	.	.	C	T	.	T	.		A	A
Hp 5	.	.	.	.	.	.	.	.	.	.	.	A	.	.	.	.	.	C	T	.	T	.		A	A
Hp 6	.	G	.	T	.	T	.	T	.	.	.	A	.	.	.	.	.	C	T	.	T	.		A	A
Hp 7	.	G	.	T	.	T	.	T	.	.	.	A	.	.	.	T	C	C	T	.	T	.		A	A
Hp 8	.	G	.	T	T	.	.	T	.	.	.	A	.	.	.	.	C	C	T	.	T	.		A	A
Hp 9	.	.	.	.	.	.	.	.	.	.	.	A	.	.	.	T	.	C	T	.	T	.		A	A
Hp 10	.	.	.	.	.	.	.	.	.	.	C	A	.	.	.	.	.	C	T	.	T	.		.	A
Hp 11	.	.	.	.	.	.	.	.	.	.	.	A	.	.	.	.	.	C	T	.	.	.		A	A
Hp 12	.	.	.	.	.	.	.	.	.	.	.	A	.	.	.	.	.	C	T	C	.	.		A	A
Hp 13	.	.	.	.	.	.	.	.	.	.	.	A	.	.	A	.	.	C	T	.	T	.		A	A
Hp 14	.	.	G	G	.	.	.	.	C	.	.	A	.	.	.	T	.	C	T	.	T	.		A	A
Hp 15	.	.	.	.	.	.	.	.	.	.	.	A	.	.	.	.	.	C	T	.	T	.		A	A
Hp 16	.	.	.	.	.	.	C	.	.	.	C	A	.	.	.	.	.	C	T	.	T	C		A	A
Hp 17	.	.	.	.	.	.	.	.	.	.	.	A	.	.	.	.	.	C	T	.	T	.		A	A
Hp 18	.	.	.	.	.	.	.	.	.	.	.	A	.	.	.	.	.	C	T	.	T	.		A	A
Hp 19	.	.	.	.	.	.	.	.	.	.	.	A	.	.	.	.	.	C	T	.	T	.		A	A
Hp 20	.	.	.	.	.	.	.	.	.	.	.	A	T	T	.	.		C	T	.	T	.		A	A
Hp 21	.	.	.	.	.	.	C	.	.	.	.	A	T	T	.	.		C	T	.	T	.		A	A
Hp 22	.	.	.	.	.	.	C	.	.	.	.	A	.	.	.	.		C	T	.	T	.		A	A
Hp 23	.	.	.	.	.	.	C	.	.	.	.	A	.	.	.	.		C	T	.	.	C		A	A
Hp 24	.	.	.	.	.	.	.	.	.	.	.	A	.	.	.	.		C	T	C	.	.		A	A
Hp 25	.	.	.	.	.	.	.	.	.	.	.	A	.	.	.	.		C	T	.	T	.		A	A
Hp 26	.	.	.	.	.	.	.	.	.	.	.	A	.	.	T	.		.	.	.	.	.		.	A
Hp 27	.	.	.	.	.	.	.	.	.	.	.	A	.	.	.	.		.	T	.	T	.		A	A
Hp 28	.	.	.	.	.	.	.	.	.	.	.	A	.	.	.	.		C	T	.	T	.		A	A
Hp 29	.	.	.	.	.	.	.	.	.	.	.	A	.	.	.	.		C	T	.	T	.		A	A
Hp 30	.	.	.	.	.	.	C	.	.	C	.	A	.	.	.	.		C	T	.	.	C		A	A
Hp 31	.	.	.	.	.	.	.	.	.	.	.	A	.	.	.	.		C	T	.	T	.		A	A
Hp 32	.	.	.	.	.	.	.	.	.	.	.	A	T	.	.	.		C	T	.	T	.		A	A
Hp 33	.	.	.	.	.	.	.	.	.	.	.	A	.	.	.	.		C	T	C	.	.	C	.	A
Hp 34	.	.	.	.	.	.	.	.	.	.	.	A	.	.	.	.		.	T	.	.	.		.	.
Hp 35	.	.	.	.	.	.	.	.	.	.	.	A	.	.	.	.		C	T	.	.	.		A	.
Hp 36	.	.	.	.	.	.	.	.	.	.	.	A	.	.	.	.		C	T	.	.	.		.	.
Hp 3	.	.	.	.	.	.	.	.	.	.	.	A	.	.	.	.		C	.	.	T	.		A	A
Hp 38	.	.	.	.	.	.	.	.	.	.	.	A	.	.	.	.		C	T	.	T	.		A	.

mt=mitochondrial, co×1 = Cytochrome c oxidase subunit 1,

* - synonymous substitution

After analyzing the mt cox1 and nad1 gene sequences, 25 polymorphic sites were identified via alignment. According to parsimonious analysis, 76% (19/25) of these polymorphic sites were informative.

### Genetic profile of *E. granulosus*

**Figure-2:**
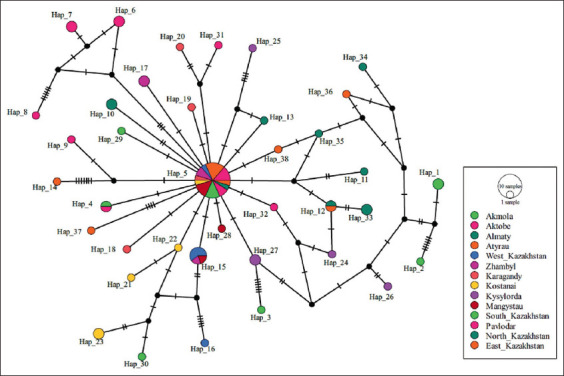
The haplotype network for the concatenated mt cox1 and nad1 genes of *Echinococcus granulosus*. The size of the circles is proportional to the frequency of each haplotype. The number of mutations separating haplotypes is indicated by dash marks. Hap=Haplotype, mt=mitochondrial, cox1=Cytochrome c oxidase subunit 1.

**Figure-3:**
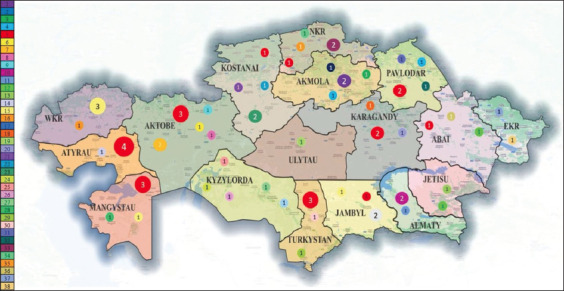
Geographical locations of haplotypes analyzed in the study. The colors of circles on the map are indicated in accordance with the haplotype, and the number of identified haplotypes is shown inside the circle [Source: https://www.qgis.org/en/site/about/].

After performing a thorough haplotype analysis, the essential focal haplotype was identified and used to configure *E. granulosus* s.s. in a circular orientation. Among the 73 samples tested, 38 unique haplotypes were identified, with the main haplotype (hp 5) accounting for 30.13% (22 out of 73) of the samples (Figures-2 and 3; Table-1).

The findings presented in Table-3 of the current report indicate that the nad1 gene exhibits an increased number of haplotype variations and nucleotide differences.

**Table-3:** DnaSP output showing diversity and neutrality indices.

DNA	n	H	hd	Tajima’s D	Fu’s Fs	FLD	p-value	FLF	p-value
E.g., *co×*1	76	5	0.352	−2.44548	−0.285	−6.26037	p < 0.01	−5.59824	p < 0.01
E.g., *nadh*1	74	34	0.843	−2.51838	−23.352	−4.86860	p < 0.02	−4.46276	p < 0.02

co×1 = Cytochrome c oxidase subunit 1, FLD=Fu and Li’s D test, FLF=Fu and Li’s F statistical test

The analysis indicates that the mt nad1 gene undergoes a higher number of mutations than the cox1 gene, as evidenced by the large number of haplotypes observed. In addition, the haplotype diversity of the nad1 gene was significantly higher, with an indicator value of 0.843 compared to cox1 of 0.352. In addition, the analysis highlights a noteworthy observation regarding the significantly high negative value of Fu’s Fs indicator for the nad1 gene (23.352). This observation could be indicative of recent population expansion or natural selection.

The analysis of haplotypes involved the examination of concatenated mt region of cox1 and nad1 marker regions. Figure-2 clearly illustrates these haplotypes, where each haplotype is differentiated by a unique color. The detailed analysis provides a comprehensive overview of the genetic variation within the studied population.

## Discussion

*E. granulosus sensu stricto* (s.s.) is widely recognized as one of the most prevalent genotypes responsible for cystic *Echinococcus* (CE) worldwide [13]. mt cox1 and NADH dehydrogenase subunit 1 (nad1) genes serve as crucial evolutionary markers to distinguish inter- and intra-specific variants. Asexual reproduction of *Echinococcus* spp. occurs during the larval stage, which can result in numerous genetic mutations and variations at both the genus and species level [14, 15].

In the present study, we performed thorough analyses to identify the specific genotypes and haplotypes of *Echinococcus* cysts that were selected from the cattle population. The aim of this study was to gain a comprehensive understanding of the genetic makeup of these cysts, which would enable us to develop more effective strategies for their management and control.

Because only a few studies have addressed the genetic diversity of the parasite in Kazakhstan [16–18], we sequenced partial mt genes of cox 1 (450 bp) and nad 1 (1200 bp) in cattle metacestodes.

Figure-2 provides a clear visual representation of the widespread distribution of haplotype 5 across the country, with the presence of haplotype 5 being detected in 10 distinct regions. Significantly, haplotype 5 is also an ancestral haplotype, which is further important to its prevalence and distribution. The information presented in Figure-2 highlights the possibility of a specific genetic variant spreading and visually represents the extensive prevalence of echinococcosis across the entire country.

Our research on genotyping revealed two distinct types of genotypes: G1 and G3. The G3 genotype was found exclusively in the Aktobe region. The genotypes G1 and G3 were classified into different haplotypes when constructing the haplotype network. Romig *et al*. [19] also observed a low level of differentiation between G1 and G3 in the haplotype network. In general, the results of the current study demonstrate low nucleotide diversity but high haplotype diversity. Two genes were negative for Tajima’s D, suggesting population expansion or purifying selection. Recent population expansion or hitchhiking in *E. granulosus* s.s. can be attributed to the significantly higher negative Fu’s Fs values in mt nad1 (23.352) than in cox1 (0.285) gene sequence comparison, which revealed the presence of rare haplotypes. Fu’s Fs test was created on the basis of haplotype distribution.

CE is prevalent in North Caucasus, Transcaucasia, Kazakhstan, Russia, Kyrgyzstan, Uzbekistan, Moldova, Ukraine, and Turkey, where livestock breeding is common [20–23]. The increase in the incidence of CE can be attributed to changes in agricultural practices following the collapse of the Soviet Union in 1991, which led to the introduction of private slaughterhouses in the backyard and subsequently to the promotion of dog infection. This disease is more common in rural areas where livestock are reared. Several factors contribute to the persistence of CE, including frequent illegal and home slaughtering of animals for food, feeding of raw offal to dogs, low public awareness of the disease, large populations of stray dogs, and poor hygiene conditions [24, 25].

## Conclusion

The findings of our research indicate that *Echinococcus* in Kazakhstan has two distinct genetic variations (G1 and G3). Our analysis showed that the G3 genotype is predominant in a specific region, whereas the G1 genotype is prevalent throughout the country. As the first study of its kind on the territory of Kazakhstan, our research is highly significant in understanding the genetic characteristics of *Echinococcus* and its potential implications for public health. In addition, our study highlights the need to continue monitoring the spread of echinococcosis throughout the country.

## Authors’ Contributions

VK: Contributed to the conception and designed the study and drafted, revised, and finalized the manuscript for submission. RU and AS: Performed DNA extraction and PCR, interpreted the data, and performed phylogenetic analysis, and drafted the manuscript for submission. SY, AA, and KJ: Collected samples and performed parasitological isolation and typing. LL and AT: Analyzed the data and performed the statistical analyses. All authors have read, reviewed, and approved the final manuscript.
